# Connecting mass-action models and network models for infectious diseases

**DOI:** 10.1371/journal.pcbi.1013373

**Published:** 2025-08-18

**Authors:** Thien-Minh Le, Jukka-Pekka Onnela

**Affiliations:** 1 Department of Mathematics, The University of Tennessee at Chattanooga, Chattanooga, Tennessee, United States of America; 2 Department of Biostatistics, Harvard T.H. Chan School of Public Health, Boston, Massachusetts, United States of America; Fundação Getúlio Vargas: Fundacao Getulio Vargas, BRAZIL

## Abstract

Infectious disease modeling is used to forecast epidemics and assess the effectiveness of intervention strategies. Although the core assumption of mass-action models of homogeneously mixed population is often implausible, they are nevertheless routinely used in studying epidemics and provide useful insights. Network models can account for the heterogeneous mixing of populations, which is especially important for studying sexually transmitted diseases. Despite the abundance of research on mass-action and network models, the relationship between them is not well understood. Here, we attempt to bridge the gap by first identifying a spreading rule that results in an exact match between disease spreading on a fully connected network and the classic mass-action models. We then propose a method for mapping epidemic spread on arbitrary networks to a form similar to that of mass-action models. We also provide a theoretical justification for the procedure. Finally, we demonstrate the application of the proposed method in the theoretical analysis of reproduction numbers and the estimation of model parameters using synthetic data based on an empirical network. The method proves advantageous in explicitly handling both finite and infinite networks, significantly reducing the computation time required to estimate model parameters for spreading processes on networks. These findings help us understand when mass-action models and network models are expected to provide similar results and identify reasons when they do not.

## Author summary

The study of infectious diseases employs two primary modeling approaches: mass-action models and network models. Mass-action models assume a fully connected graph for interactions, while network models typically feature more complex, less connected graphs. The relationship between these two modeling approaches is unclear. To address this knowledge gap, we propose a method that allows us to match the mass-action and network models when the graph is fully connected. We also introduce a mapping, which makes use of the structure of the network, to transform epidemic spread on arbitrary networks into models that are analogous to mass-action models. Simulation results indicate that using the proposed mapping method reduces computational time by a factor of approximately 30 with little loss in accuracy. The proposed approach has been validated theoretically and through simulations, showing its potential to enhance approximate Bayesian parameter estimation. Overall, these findings offer valuable insights into disease dynamics and suggest new ways to integrate traditional mass-action results into network analyses.

## 1. Introduction

Understanding disease spread is crucial for providing accurate predictions of disease outbreaks and for gaining greater insight into prevention strategies. Infectious disease modeling has served as a potent tool for this endeavor for centuries, with the earliest work dating back to the work of Bernoulli in 1760 [1]. Compartmental mass-action models are the most common type of modeling approach, and they are frequently used to study different influenza strains. This modeling assumes that all individuals are well-mixed [2]. Its advantage is that it is simple to use and has a well-established theoretical foundation for different disease properties, while still producing accurate predictions for multiple types of diseases, particularly influenza [3,4]. Sexually transmitted diseases such as monkeypox, human immunodeficiency virus (HIV), and human papillomavirus (HPV) are challenging for mass-action models as infected individuals transmit diseases to their neighbors only through a sexual network. Since population structure is naturally represented as a network, there have been numerous investigations into the spread of disease on networks in the last two decades [5–10].

Despite the fact that network epidemiology has received a lot of attention from the research community, most of the work has focused on deriving solutions for spreading processes on static or dynamic networks or understanding the effects of network topology characteristics on spreading process outcomes [5–7,11]. Surprisingly, there are few studies on the connection of network models and mass-action models. Understanding the connection between the two model families is critical because it will allow researchers to see the effect of network topology on the spreading process and could open up new avenues for making use of well-established results from classic models. Levin and Durrett (1996) highlighted the similarity between the SIR network-based model and the SIR mass-action model, with the primary distinction lying in the interaction between the susceptible and infectious compartments [12]. Other researchers extended the network-based model by developing approaches to study spreading processes on networks within a more generalized framework, including directed, semi-directed, and message-passing dynamics. These studies also confirmed that the results of the classic mass-action model emerge as a special case of the network-based model in the limit of large networks [13–17]. The first attempt to make use property of a network-based model to fit in a classic model was the work of Keeling [18]. Keeling (2005) proposed a modified mass-action model to fit the network model’s predictions. The transmission rate of the modified mass-action model was defined to be a function of certain network characteristics (the average degree and the ratio of triangles to triples). Kenah (2010) introduced a contact interval approach and used simulations to illustrate the limitations of naively applying the mass-action model when estimating the reproduction number $R_{0}$ generated from a network-based model [19]. Malloy et al. (2021) used different simulation settings to investigate the influence of the mass-action model and the network model on the effectiveness of prevention strategies [20]. Recently, Rempala demonstrated that the Poisson SIR network model approximates the classic SIR model as the mean degree of the network increases [21]. Despite these efforts, the existing studies mostly focus on the large sample behavior of networks, and the explicit relation between the two models was not spelled out clearly.

The purpose of this work is to bridge the gap in the literature by relating the two models. The primary distinction between mass-action models and network-based models lies in their graph structures, as the implicit contact graphs of classic mass-action models are always fully connected, whereas the graphs of network-based models are usually not. In order to connect these two models, we will first study the behavior of epidemic spread on fully connected graphs. There are several ways to define the spreading process on networks, including the Gillespie, degree infectivity, and unit infectivity methods [7,22,23]. The classical Gillespie method is a continuous-time, event-driven stochastic simulation in which infections occur at a time. The infected node is chosen from susceptible nodes with infected neighbors using weighted sampling, and nodes with more infected neighbors have higher infection rates [7]. A discrete-time version can be adapted from this formulation by applying the same rate-based infection logic (see Algorithm 1 in the S1 File). In the unit infectivity method, at each time step, each infected node randomly chooses a neighbor, and then transmits the disease to the chosen neighbor with a fixed probability [23]. Unlike the unit infectivity method, in the degree infectivity method, at each time step each infected node transmits the disease to each of its neighbors with a fixed probability [22]. Degree infectivity is the most commonly used spreading method in networks and is similar to bond percolation on networks. More precisely, when infectiousness does not vary and the infectious period is fixed, the degree infectivity process reduces exactly to classical bond percolation on the network [24]. Under these conditions, a mass‑action model on a fully mixed (Erdős–Rényi) network can also be formulated as bond percolation. The pseudo codes in Algorithm 1,2,3 in S1 File describe how these spreading methods work. In this work, we adopt a stochastic discrete-time modeling framework, in which infection counts remain integer-valued. While classical mass-action models are often formulated in continuous time and can produce non-integer expected infection counts, the discrete-time formulation serves as a natural foundation for bridging mass-action and network-based models. This choice is motivated by both practical and theoretical considerations: when the time step is sufficiently small, the discrete-time model closely approximates continuous-time dynamics, and most empirical epidemic data—such as case counts—are recorded at regular intervals (e.g., daily or weekly). These properties make the discrete-time framework particularly convenient for inference and model comparison. To connect the two models, the spreading process on fully connected graphs should yield identical results, regardless of how the spreading rule is defined. In the discrete-time framework, the unit infectivity method, which restricts each infected node to a single attempted transmission event per time step, and the discrete-time adaptation of the Gillespie method, which allows only one infection event per time step, will not yield the same result as the mass-action model on fully connected graphs. Therefore, we will only consider degree infectivity and refer to it as the conventional network spreading method. Under the conventional network spreading method, however, the number of infections on fully connected graphs is always less than the number of infections under the mass-action model [7]. We first propose a rule for network propagation that eliminates this discrepancy. Then, based on the proposed spreading rule, we present approaches to employ network topology to adapt the mass-action model to capture the spread on networks. We also provide theoretical justifications to support our method. Finally, using simulation and synthetic data, we show the merits of the proposed method in studying epidemics on networks.

The structure of the paper is as follows. In Section 2.1, we discuss the classic mass-action models and our proposed spreading process on networks. Sections 2.2 and 2.3 provide the approximation procedure for the proposed spreading process and offer theoretical justifications for it. Section 3 provides results demonstrating the merits of the proposed method in studying epidemics on networks. In particular, Section 3.1 demonstrates that the proposed method enables the calculation of reproduction numbers on networks in a manner similar to the mass-action model. It also provides theoretical results comparing the early dynamics of the epidemic between the network and mass-action models. Section 3.2 shows the merits of employing the proposed methods for analyzing epidemics on networks. We highlight the advantages of the proposed approach to significantly reduce computational time with minimal loss of accuracy when studying epidemics on networks. Additionally, Section 3.3 provides a quantitative answer to a fundamental question regarding the necessity of network-based models in epidemic studies: If network information were readily available, how valuable would it be compared to simply using the mass-action model? Finally, Section 4 discusses our contribution and possible directions for future research.

## 2. Materials and methods

### 2.1 Mass-action models and network models

#### 2.1.1 Mass-action models.

Mass action models are the most common model types used in infectious disease epidemiology due to their simplicity. The model makes the fundamental assumption of homogeneous mixing of all individuals, implying that the contact network is fully connected: any infectious individual can potentially transmit infection to any susceptible individual. This section examines the Susceptible-Infected (SI), Susceptible-Infected-Recovered (SIR), and Susceptible-Infected-Treated-AIDS-Death (SITAD) processes. The first two processes, SI and SIR, are frequently used for influenza. The SITAD model here is a simplified version of the model used in Hove-Musekwa et. al. [25] to study Human immunodeficiency virus infection and acquired immune deficiency syndrome (HIV/AIDS).

**Fig 1:**
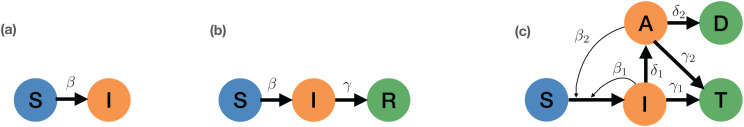
Three different spreading processes: (a) the SI spreading process, (b) the SIR spreading process, and (c) the SITAD spreading process.

**SI process**: In the SI process, at a given time, the population is divided into two mutually exclusive compartments: susceptible and infected. Suppose N is the size of the population. Let S and I denote the number of susceptible and infected individuals, respectively (S+I=N). Suppose that the model parameter is θ=β, where β is the transmission rate. Its dynamic states evolve as in Fig 1a.

Let us denote the status of its population at time t is ${𝐗}_{t}=[S_{t},I_{t}]$. Using the tau leaping method by Gillespie [26], the status of its population at time (t+τ) evolves as ${𝐗}_{t+\tau }={𝐗}_{t}+Y_{1}(h_{1}({𝐗}_{t}tau)\nu_{1}$. Here, $\nu_{1}=[-1,1{]}^{T}$ is the transition vector, $h_{1}({𝐗}_{t})=\beta \frac{S_{t}I_{t}}{N}$ is the population level hazard, and $Y_{1}(h_{1}({𝐗}_{t}tau)$ is Poisson distributed with mean $h_{1}({𝐗}_{t}tau$. By choosing τ=1, which represents the change in population status after each time unit, the dynamic epidemic in the population evolves from ${𝐗}_{t}=[S_{t},I_{t}]$ to ${𝐗}_{t+1}=[S_{t+1},I_{t+1}]$ by the transformation ${𝐗}_{t+1}={𝐗}_{t}+Y_{1}(h_{1}({𝐗}_{t}))\nu_{1}$. In particular, the evolving process can be described in the system of equations (1) below:


$$\left\{ \begin{array}{c} S_{t+1}=S_{t}-Y_{1,t}\\ I_{t+1}=I_{t}+Y_{1,t}, \end{array}\right.$$ (1)

where $Y_{1,t}=Y_{1}(h_{1}({𝐗}_{t}))$ is Poisson distributed with mean $h_{1}({𝐗}_{t})$.

**SIR process**: The population for the SIR process at a given time is divided into three mutually exclusive compartments: susceptible, infected, and recovered. Suppose N is the size of the population. Let S,I,R denote the number of susceptible, infected, and recovered individuals, respectively (S+I+R=N). Suppose that the model parameter is θ=(β,γ), where β is the transmission rate and γ is the recovery rate. Its dynamic states evolve as in Fig 1b.

Denote the status of its population at time t is ${𝐗}_{t}=[S_{t},I_{t},R_{t}]$. The tau leaping method tells us the status of its population at time (t+τ) evolved as ${𝐗}_{t+\tau }={𝐗}_{t}+\sum_{j=1}^{2}Y_{j}(h_{j}({𝐗}_{t}tau)\nu_{j}$, where $\nu_{1}=[-1,1,0{]}^{T}$ and $\nu_{2}=[0,-1,1{]}^{T}$ are the transition vectors, and $Y_{j}(h_{j}({𝐗}_{t}tau)$, for j=1,2, are random variables. Here $Y_{j}(h_{j}({𝐗}_{t}tau)$ Poisson distributed with means $h_{j}({𝐗}_{t}tau$, where $h_{1}({𝐗}_{t})=\beta \frac{S_{t}I_{t}}{N}$ and $h_{2}({𝐗}_{t})=\gamma I_{t}$ are population level hazards. Let τ=1, which represents the change in population status after each time unit, the dynamic epidemic in the population evolves from ${𝐗}_{t}=[S_{t},I_{t},R_{t}]$ to ${𝐗}_{t+1}=[S_{t+1},I_{t+1},R_{t+1}]$ by the transformation ${𝐗}_{t+1}={𝐗}_{t}+\sum_{j=1}^{2}Y_{j}(h_{j}({𝐗}_{t}))\nu_{j}$. More specifically, the shift from time t to time t+1 of the SIR process can be described as the system of equations (2) below.


$$\left\{ \begin{array}{c} S_{t+1}=S_{t}-Y_{1,t}\\ I_{t+1}=I_{t}+Y_{1,t}-Y_{2,t}\\ R_{t+1}=R_{t}+Y_{2,t}, \end{array}\right.$$ (2)

where $Y_{1,t}=Y_{1}(h_{1}({𝐗}_{t}))$, $Y_{2,t}=Y_{2}(h_{2}({𝐗}_{t}))$ are Poisson distributed with means $h_{1}({𝐗}_{t}),h_{2}({𝐗}_{t})$, respectively.

**SITAD process**: We consider a simplified version of the HIV/AIDS model of Hove-Musekwa et. al. [25]. In this model, at a given time, the population’s state is divided into five mutually exclusive compartments: susceptible (S), HIV positive (I), AIDS (A), treated (T), and deceased (D) (S+I+A+T+D=N). Its dynamic state evolves as in Fig 1c, where the model parameter $\theta =(\beta_{1},\beta_{2},\gamma_{1},\delta_{1},\gamma_{2},\delta_{2})$, $\beta_{1}$ is the transmission rate of HIV, $\beta_{2}$ is the transmission rate of AIDS, $\gamma_{1}$ is the treatment rate of HIV, $\delta_{1}$ is the AIDS progression rate of HIV, $\gamma_{2}$ is the treatment rate of AIDS, and $\delta_{2}$ is the death rate of AIDS.

Let the status of its population at time t be ${𝐗}_{t}=[S_{t},I_{t},T_{t},A_{t},D_{t}]$. Using the tau leaping method, the dynamic epidemic of the population at time step (t+τ) evolves as ${𝐗}_{t+\tau }={𝐗}_{t}+\sum_{j=1}^{5}Y_{j}(h_{j}({𝐗}_{t}tau)\nu_{j}$, where $\nu_{1}=[-1,1,0,0,0{]}^{T}$, $\nu_{2}=[0,-1,1,0,0{]}^{T}$, $\nu_{3}=[0,-1,0,1,0{]}^{T}$, $\nu_{4}=[0,0,1,-1,0{]}^{T}$, and $\nu_{5}=[0,0,0,-1,1{]}^{T}$ are the transition vectors. And $Y_{j,t}=Y_{j}(h_{j}({𝐗}_{t}))$ are Poisson distributed with means $h_{j}({𝐗}_{t}tau$, for j=1,…,5. Here, $h_{1}({𝐗}_{t})=(\beta_{1}I_{t}+\beta_{2}A_{t})S_{t}/N$, $h_{2}({𝐗}_{t})=\gamma_{1}I_{t}$, $h_{3}({𝐗}_{t})=\delta_{1}I_{t}$, $h_{4}({𝐗}_{t})=\gamma_{2}A_{t},$ and $h_{5}({𝐗}_{t})=\delta_{2}A_{t}$ are population level hazards. Let τ=1, which represents the change in population status after each time unit, the dynamic epidemic in the population evolves from ${𝐗}_{t}=[S_{t},I_{t},T_{t},A_{t},D_{t}]$ to ${𝐗}_{t+1}=[S_{t+1},I_{t+1},T_{t+1},A_{t+1},D_{t+1}]$ by the transformation ${𝐗}_{t+1}={𝐗}_{t}+\sum_{j=1}^{5}Y_{j}(h_{j}({𝐗}_{t}))\nu_{j}$. In particular, the shifted from time t to t+1 of the SITAD process is described as in the system of equations (3) as below.


$$\left\{ \begin{array}{c} S_{t+1}=S_{t}-Y_{1,t}\\ I_{t+1}=I_{t}+Y_{1,t}-Y_{2,t}-Y_{3,t}\\ T_{t+1}=T_{t}+Y_{2,t}+Y_{4,t}\\ A_{t+1}=A_{t}+Y_{3,t}-Y_{4,t}-Y_{5,t}\\ D_{t+1}=D_{t}+Y_{5,t}, \end{array}\right.$$ (3)

where $Y_{j,t}=Y_{j}(h_{j}({𝐗}_{t}))$, for j=1,…,5, are Poisson distributed with means $h_{j}({𝐗}_{t})$.

#### 2.1.2 Network models and the proposed spreading process.

The graphs of the network-based model are usually not fully connected, so the spreading process depends on network topology. For simplicity, we consider a given fixed network G with a known initial single infected node.

The spreading process on networks occurs between infected and susceptible individuals, where at each time step each infected node has a fixed probability of transmitting the disease to each of its susceptible neighbors. Note that the meaning of the transmission parameter of the spreading process on networks differs from that of the transmission parameter in the mass-action model [22].

In a discrete-time network spreading process, β represents the per-time-step probability that an infected node transmits infection to one of its susceptible neighbors (In continuous-time formulations, β is typically treated as a transmission rate, with the per-time-unit transmission probability approximated by $1-e^{-\beta }\approx \beta$ when β is small). In mass-action models, the transmission parameter $\beta_{m}$ represents the average number of effective contacts per unit time between infectious and susceptible individuals that result in new infections. Since there are S susceptible individuals out of a total of N individuals in the population, the probability of randomly selecting a susceptible individual from the entire population is S/N. Therefore, the total number of new infections caused by each infected individual is $\beta_{m}S/N$. Therefore, on a graph with N fully connected nodes, the transmission parameter $\beta_{m}$ of the mass-action model corresponds to the transmission rate $\beta =\beta_{m}/N$ in the network model. The same distinction was also pointed out between the contact interval distribution and scaled contact interval distribution as in [19].

To evaluate the connection between the network model and the traditional mass-action model, we first examine if there is any discrepancy in the number of infectious each model generates over time in a fully connected graph. Because the topologies of the populations under the two models are identical, we expect that the number of infections generated from each model will be the same. However, the spreading process on a fully connected network results in fewer infections than the mass-action model for finite networks, with the gap between the two models closing only as the network size N→∞ [7]. The gap is intuitive as the nature of new infections formed for each model is different. Under the mass-action model, at each time step, the model generates a total of new infections. Due to the homogeneous mixing assumption, any susceptible individual can be infected, regardless of their location. In mass-action models, each time step can be understood as a two-stage process: first, the total number of new infections is determined at the global (network) level, and then these infections are randomly assigned to susceptible nodes at the local level.

On the other hand, under the conventional spreading rule of the network-based model, at each time step, each infected node will spread the disease to its susceptible neighbors with a given probability. Since multiple infected neighbors may attempt to infect the same susceptible node during each time step, the total number of new infections at each time step can only be determined after the entire spreading process from all infected nodes is completed.

So, under the network-based model, at each time step, the transmission flow goes from the generation of infections from each infected individual (“local level”) to the total new infections (“global level”). Therefore, the network model may produce a lower number of infections when, at the local level, one susceptible node may be infected by two or more of its infected neighbors.

This discrepancy is more problematic when the probability that at least one susceptible node will be infected by more than one infectious neighbor at time t increases, causing infected nodes in a sense to “compete” for susceptible nodes. This phenomenon was also discussed in the work of Kenah and his co-authors [27]. Therefore, as long as this discrepancy persists, the gap between the two models persists. Thus, to bridge the mass-action models and network models, we need to propose a new spreading rule that can close this gap when the graph is fully connected.

The proposed spreading rule adopts the transmission parameter as defined in the mass-action model. Specifically, for each infected node, the transmission rate β represents the average number of contacts with randomly selected susceptible individuals that result in new infections per unit time. For an infected node i, its local “bubble” consists of $k_{i}+1$ individuals, including node i itself and its $k_{i}$ neighbors. The probability that a randomly selected individual from this bubble is susceptible is $S_{i}/(k_{i}+1)$, where $S_{i}$ denotes the number of susceptible individuals in the bubble. The use of $k_{i}+1$ in the denominator ensures a natural connection between mass-action and network-based models, particularly in finite-size networks. To illustrate, one may imagine that each infected node produces a fixed number of pathogens per unit time, which is then shared uniformly among the members of its local bubble, including the node itself. Consequently, the expected number of new transmissions caused by an infected node i is given by $\beta S_{i}/(k_{i}+1)$.

Under the proposed spreading rule, at each time step, the total transmission rate is computed first, and the number of new infections is then generated based on the total transmission rate. New infections are assigned to at-risk nodes (susceptible neighbors of infected nodes) using a weighted random sample, where the weight of each at-risk node is proportional to the number of infected neighbors. More specifically, let ${\text{I}}_{t}$ be the set of infected nodes at time t, $S_{i,t}$ be the number of susceptible neighbors of node i at time t, and $S_{t}^{*}$ be the number of at-risk nodes at time t. The total transmission rate at time t+1 is calculated as $h_{1}({𝐗}_{t})=\beta \sum_{i\in \text{I}(t)}\frac{S_{i,t}}{k_{i}+1}$. The number of new infections $Y_{1,t}$ is generated from $\text{Binom}\left(S_{t}^{*},h_{1}({𝐗}_{t})/S_{t}^{*}\right)$, where $S_{t}^{*}$ is the number of at-risk nodes at time t. This Binomial distribution approximates a Poisson distribution with mean $h_{1}({𝐗}_{t})$ when $S_{t}^{*}$ is large. The new infections are then randomly allocated among the at-risk nodes based on their weights (see Algorithm 4 in S1 File for more details on the proposed SI spreading rule).

Under the proposed spreading process, when the network is fully connected, susceptible nodes and at-risk nodes are the same, i.e., $S_{t}^{*}=S_{t}$. Therefore, at time t, the total transmission rate is $\beta I_{t}S_{t}/N$, and new infections are generated from Binomial$\left(S_{t},\beta I_{t}/N\right)$ given that $\beta I_{t}/N<1$. Our proposed procedure allows for a good match with the mass-action model as long as Binomial$\left(S_{t},\beta I_{t}/N\right)$ is a good estimate for Poisson$\left(\beta S_{t}I_{t}/N\right)$. It should be noted that the proposed SI spreading rule will yield an exact match with the SI mass-action model in (1) for any network size if one chooses to model $Y_{1,t}$ using a Binomial distribution instead of the Poisson distribution.

**Fig 2:**
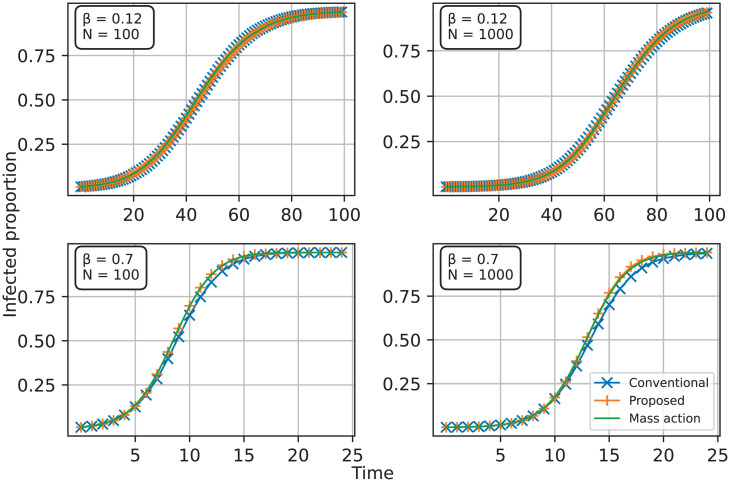
Comparison of the SI process of the proposed network spreading rule (Proposed) on fully connected graphs, the conventional network spreading rule (Conventional), and the mass-action model (Mass-action) with the transmission parameter β = 0.12 with 100 nodes (top left) and 1000 nodes (top right), and β = 0.7 on fully connected graphs with 100 nodes (bottom left) and 1000 nodes (bottom right).

Fig 2 displays the average proportion of infections over time for an SI process of the proposed spreading rule, the conventional network spreading rule, and the mass-action model on fully connected graphs. Here we consider four cases: graphs of 100 nodes and 1000 nodes with the transmission parameter β=0.12 (top left and top right), and graphs of 100 nodes and 1000 nodes with the transmission parameter β=0.7 (bottom left and bottom right). The average is taken over 200 stochastic realizations. As expected, as the transmission parameter is small, the different spreading rules are hard to distinguish. But when the transmission parameter is large, the proposed spreading rule still precisely matches the mass-action model, but the conventional spreading rule on the network underestimates the number of infections relative to the mass-action model. Although this discrepancy of all spreading rules will be removed for large networks as N→∞, the proposed spreading rule helps to alleviate the discrepancy for all cases, especially for finite networks. Therefore, the proposed spreading rule is an excellent choice for bridging the mass-action models and network-based models.

### 2.2 Approximations to the proposed spreading process

The most straightforward strategy for studying epidemic spread on network is to use simulation. Despite the fact that this method provides us with many insights into various disease-spreading processes in a variety of settings, it does not give us a thorough theoretical understanding of the spreading process. Much effort has been dedicated to investigating the solution of the spreading process using the conventional network spreading rule. However, most approaches are for large populations, which provide approximate solutions for large networks [15,28–31]. Recently, efforts applying dynamical survival analysis studying network epidemics helped to encompass both mass-action models and network-based models for the configuration model [32–35]. Although these approaches provide great insights into the spreading process on networks, they still provide limited insights into how network models and mass-action models are related, especially for finite-size networks.

In the following, we present another approach for a better understanding of the relationship between network models and mass-action models. The main idea is to set up a system of equations that are analogous to those of the mass-action model while also taking into account the topology of the network. For example, for the SI process, we aim to replace βIS/N with Iβ(G), where β(G) is a function that contains information about network topology.

#### 2.2.1 The modified SI process.

**Fig 3:**
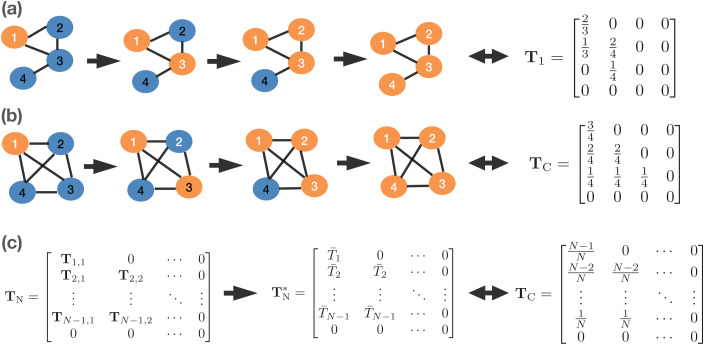
The connection in transmission rate between network model and mass-action model. **(a)** Transmission order on a network of four nodes and its corresponding transmission matrix. **(b)** Transmission order on a complete network of four nodes and its corresponding transmission matrix. **(c)** Connection of the network transmission matrix and the transmission matrix corresponding to the mass-action model.


Consider an SI process with transmission rate β, starting with one infected node and spreading the disease to all over the network. If the order of infections is known, we introduce a transmission matrix $𝐓=[{𝐓}_{i,j}{]}_{N\times N}$ based on network topology. Element (i,j) of the transmission matrix 𝐓, ${𝐓}_{i,j}$, represents the transmission rate caused by network topology of node j when the network has i infected nodes. Here ${𝐓}_{i,j}=S_{i,j}/(k_{j}+1)$, where $S_{i,j}$ is the number of susceptible neighbors of node j when there are i infected nodes in the network, and $k_{j}$ is the degree of node j. The summation of row i in the transmission matrix, $\sum_{j=1,N}{𝐓}_{i,j}$, gives the overall spreading rate caused by network topology when there are i infected nodes in the network. Thus, the overall transmission rate when the network has i infected nodes is $\beta \sum_{j=1,N}{𝐓}_{i,j}$. Since reordering the column of the transmission matrix 𝐓 will not change the overall transmission rate (the row sum), for simplicity, we rearrange the columns of the transmission matrix in the order of infections and use this rearranged matrix as our transmission matrix 𝐓. Fig 3a demonstrates the transmission matrices corresponding to the infection order (1,3,2,4) for the incomplete network with 4 nodes. Here the transmission matrix ${𝐓}_{1}$ results from reordering the columns of the original transmission matrices from (1,2,3,4) to (1,3,2,4) as

$$\begin{array}{c} {𝐓}_{1}=[\begin{array}{cccc} \frac{2}{3} & 0 & 0 & 0\\ {}[3pt]\frac{1}{3} & 0 & \frac{2}{4} & 0\\ {}[3pt]0 & 0 & \frac{1}{4} & 0\\ {}[3pt]0 & 0 & 0 & 0 \end{array}]\to {𝐓}_{1}=[\begin{array}{cccc} \frac{2}{3} & 0 & 0 & 0\\ {}[3pt]\frac{1}{3} & \frac{2}{4} & 0 & 0\\ {}[3pt]0 & \frac{1}{4} & 0 & 0\\ {}[3pt]0 & 0 & 0 & 0 \end{array}] \end{array}$$

Similarly, Fig 3b represents the transmission matrices ${𝐓}_{\text{C}}$ corresponding to the infection order (1,3,2,4) for the complete network with 4 nodes. Here the transmission matrix ${𝐓}_{\text{C}}$ results from reordering the columns of the original transmission matrices from (1,2,3,4) to (1,3,2,4) as


$$\begin{array}{c} {𝐓}_{\text{C}}=[\begin{array}{cccc} \frac{3}{4} & 0 & 0 & 0\\ {}[3pt]\frac{2}{4} & 0 & \frac{2}{4} & 0\\ {}[3pt]\frac{1}{4} & \frac{1}{4} & \frac{1}{4} & 0\\ {}[3pt]0 & 0 & 0 & 0 \end{array}]\to {𝐓}_{\text{C}}=[\begin{array}{cccc} \frac{3}{4} & 0 & 0 & 0\\ {}[3pt]\frac{2}{4} & \frac{2}{4} & 0 & 0\\ {}[3pt]\frac{1}{4} & \frac{1}{4} & \frac{1}{4} & 0\\ {}[3pt]0 & 0 & 0 & 0 \end{array}]. \end{array}$$

Let us denote $\sum_{k=1}^{N}{𝐓}_{I_{t},k}=I_{t}{\overset{―}{T}}_{I_{t}}$. The behavior of the spreading process on the network using the transmission matrix 𝐓 after each time unit then can be described by


$${𝐗}_{t+1}={𝐗}_{t}+Y_{1}\left(h_{1}\left({𝐗}_{t}\right)\right)\nu_{1},$$ (4)

where ${𝐗}_{t+1}=(S_{t+1},I_{t+1}),{𝐗}_{t}=(S_{t},I_{t}),\nu_{1}=[-1,1{]}^{T}$, and $Y_{1}(h_{1}({𝐗}_{t}))$ is Poisson distributed with mean $h_{1}({𝐗}_{t})$ = $\beta I_{t}{\overset{―}{T}}_{I_{t}}$.

Compared to the SI mass-action model, it is apparent that the SI process on networks is controlled by network topology through the transmission matrix 𝐓, where the mass-action transmission rate $\beta I_{t}S_{t}/N$ is replaced by $\beta I_{t}{\overset{―}{T}}_{I_{t}}$. Therefore, the transmission matrix 𝐓 encompasses all the network information related to the spreading process. Instead of directly simulating the spread of the disease on the network, using the transmission matrix allows us to study the spreading process more quantitatively. The relationship between a network model and the mass-action model is depicted in Fig 3c. In contrast to the transmission matrix of the conventional mass-action model, which is always ${𝐓}_{\text{C}}$, the transmission matrix of the network model will vary according to the network topology and takes the form ${𝐓}_{\text{N}}$. Under our procedure, the actual transmission matrix ${𝐓}_{\text{N}}$ is approximated by ${𝐓}_{\text{N}}^{*}$, where non-zero elements in each row represent the average infection rate at that time.

Let $I_{𝐓,t}$ denote the sequence of the number of infected nodes based on the transmission matrix 𝐓 at time t. Denote $E(I_{𝐓,1}^{+})=E(I_{𝐓,1}|I_{𝐓,0}=1)$, ⋯, $E(I_{𝐓,k}^{+})=E(I_{𝐓,k}|I_{𝐓,k-1}=E(I_{𝐓,k-1}^{+}))$. Therefore, the average number of infected nodes from time 1 to K in the network is $E(I_{1}^{+}),\cdots ,E(I_{K}^{+})$. Similarly, the average number of susceptible nodes from time 1 to K in the network is $E(S_{1}^{+}),\cdots ,E(S_{K}^{+})$.

Lemma 2.1 below tells us that the proposed network spreading process and the modified network spreading process have the same average realization.

**Lemma 2.1** When the order of infections is known, the spreading process based on equation (4) has the same average realization as the proposed SI process on the network.

If the order of infections is unknown, we can obtain the infection order by a random sample. In this approach, each newly infected node is incrementally updated by sampling at-risk nodes based on their risk weights (see Algorithm 7 in S1 File for details). Then, using the same procedure as before, we can construct the corresponding transmission matrix for each sample and calculate the number of infections. Finally, the average number of infections is obtained by averaging the number of infections corresponding to the sampled infection order sequences. This sampling scheme has the following rationale. Consider the network G with N nodes and a known first infected node; there are at most (N−1)! possible infection order sequences. Assuming that we obtained m infection order sequences using the sampling approach, let ${𝐓}_{i}$ denote the transmission matrix corresponding to the infection order sequence i. The average realization of the number of infections can then be approximated by the average realization of the number of infections from transmission matrices ${𝐓}_{i}$. Therefore, as the sample size m grows, the average number of infections based on transmission matrices ${𝐓}_{i}$ will converge to the average realization of the spreading process on the network.

*Remark 1:* Although the sampling approach used to obtain the m-infection order sequence is similar to directly simulating epidemics on a network, the transmission matrix derived from this sampling procedure provides deeper insights into how the network topology influences the transmission rate. For certain networks, such as complete networks, k-star networks, and cycle networks, the transmission matrix remains unchanged. This allows us to examine the impact of network topology on the spreading process through the transmission matrix, an insight that would be difficult to uncover through direct simulation alone. This represents a key advantage of the proposed method.

*Remark 2:* There is a close similarity between the method of obtaining the infection order sequence in the proposed approach (Algorithm 7 in S1 File) and the use of epidemic percolation networks (EPNs) to simulate the spreading process in networks, as described in Kenah and Miller (2011) [36]. EPNs provide a flexible and general framework for modeling stochastic epidemic processes, including settings with heterogeneous infectiousness and susceptibility, as well as hybrid models that combine network-based and mass-action transmission mechanisms. While the transmission sequence in EPNs is typically generated through a simple random procedure, the proposed approach uses a weighted sampling method where each at-risk node’s probability of infection is proportional to its number of infectious neighbors. Although the two approaches may be equivalent under certain assumptions, they differ in formulation and intended use: EPNs are often employed to study final epidemic size, whereas the proposed method is intended to establish connections between network-based and mass-action models. Exploring formal connections between these frameworks is a promising direction for future work.

#### 2.2.2 The modified SIR process.

Similarly to the SI process, we first consider the infection order of all nodes when it is known, and when it is unknown, we use the same sampling procedure as above. For the SIR process, due to the effect of network topology, the recovery of an infected node in one location may result in a different number of at-risk nodes (susceptible neighbors of infected nodes) compared to when an infected node recovers in a different location. Therefore, in the SIR process, the same number of recovered nodes may result in a different number of at-risk nodes. If recovery occurs at random, the Binomial approximation cannot be used. Therefore, the exact match in terms of the number of infections will only be feasible if the recovery order is determined by the length of time a node was infected. As random recovery is a common assumption, we will focus on this case. As the number of at-risk nodes is unattainable, we can generate the number of newly infected nodes at each time step using the Poisson distribution. For a given infection order sequence, we can extract the transmission matrix 𝐓 corresponding to the case in which there is no recovery during transmission. Denote ${\overset{―}{T}}_{H}=\sum_{k=1}^{N}{𝐓}_{H,k}/H,H=I+R$. The modified SIR spreading process based on the transmission matrix 𝐓 can now be updated as


$$\begin{array}{c} {𝐗}_{t+1}={𝐗}_{t}+\sum {\mathrm{\backslash nolimits}}_{j=1}^{2}Y_{j}\left(h_{j}\left(𝐗(t)\right)\right)\nu_{j}, \end{array}$$ (5)

where ${𝐗}_{t+1}=(S_{t+1},I_{t+1},R_{t+1}),{𝐗}_{t}=(S_{t},I_{t},R_{t}),\nu_{1}=[-1,1,0{]}^{T},\nu_{2}=[0,-1,1{]}^{T}$; $Y_{1}(h_{1}({𝐗}_{t}))$ and $Y_{2}(h_{2}({𝐗}_{t}))$ are Poisson distributed with means $h_{1}({𝐗}_{t})=\beta I_{t}{\overset{―}{T}}_{H}$ and $h_{2}({𝐗}_{t})=\gamma I_{t}$, respectively. Note that the Poisson approximation is accurate if the network is dense or if the transmission rate is small compared to the network density.

Let $I_{𝐓,t}$ and $R_{𝐓,t}$ denote the sequence of the number of infected nodes and recovered nodes based on the transmission matrix 𝐓 at time t, respectively. Denote $E(I_{𝐓,1}^{+})=E(I_{𝐓,1}|I_{𝐓,0}=1,R_{𝐓,0}=0)$, $E(R_{𝐓,1}^{+})=E(R_{𝐓,1}|I_{𝐓,0}=1,R_{𝐓,0}=0)$, ⋯, $E(I_{𝐓,k}^{+})=E(I_{𝐓,k}|I_{𝐓,k-1}=E(I_{𝐓,k-1}^{+}),R_{𝐓,k-1}=E(R_{𝐓,k-1}^{+}))$, $E(R_{𝐓,k}^{+})=E(R_{𝐓,k}|I_{𝐓,k-1}=E(I_{𝐓,k-1}^{+}),R_{𝐓,k-1}=E(R_{𝐓,k-1}^{+}))$. Then the average realization of the number of nodes in each state from time 1 to K on the network is $E(Y_{1}^{+}),\cdots ,E(Y_{K}^{+}),$ where Y is one of the states S,I,R.

The following lemma shows that the two spreading processes have the same average realization.

**Lemma 2.2** If the infection order sequence is known, the spreading process based on equation (5) has the same average realization as the SIR process on the network.

#### 2.2.3 The modified SITAD process.

The SITAD process on networks starts from an initially infected node and then spreads to cause new HIV infections with rate $\beta_{1}$. Among those infected with HIV, some progress to AIDS and some get treated. Individuals with AIDS spread the disease and cause new HIV infections with rate $\beta_{2}$. Among individuals with AIDS, some will get treated and some will die. The risk weight of each at-risk node in the SITAD spreading process is determined by $w=\beta_{1}\times \text{numberHIVneighbors}+\beta_{2}\times \text{numberAIDSneighbors}$. Similarly to the SIR process, we consider the case where AIDS progression, treatment, and death happen at random, and the infection order is known. Let 𝐓 be the transmission matrix corresponding to the infection order sequence. The modified SITAD spreading process based on the transmission matrix 𝐓 can now be updated as follows:

Denote ${\overset{―}{T}}_{H}=\sum_{k=1}^{N}{𝐓}_{H,k}/H,H=I+T+A+D$. Here H is the total number of people with HIV. The modified SITAD spreading process based on the transmission matrix 𝐓 can now be updated as


$${𝐗}_{t+1}={𝐗}_{t}+\sum_{j=1}^{5}Y_{j}\left(h_{j}\left(𝐗(t)\right)\right)\nu_{j},$$ (6)

where ${𝐗}_{t+1}=[S_{t+1},I_{t+1},T_{t+1},A_{t+1},D_{t+1}]$, ${𝐗}_{t}=[S_{t},I_{t},T_{t},A_{t},D_{t}]$, $\nu_{1}=[-1,1,0,0,0{]}^{T},$ $\nu_{2}=[0,-1,1,0,0{]}^{T},$ $\nu_{3}=[0,-1,0,1,0{]}^{T},$ $\nu_{4}=[0,0,1,-1,0{]}^{T},$ $\nu_{5}=[0,0,0,-1,1{]}^{T}$. $Y_{j}(h_{j}({𝐗}_{t}))$, for j=1,…,5, are Poisson distributed with means $h_{j}({𝐗}_{t})$. Here, $h_{1}({𝐗}_{t})=(\beta_{1}I+\beta_{2}A){\overset{―}{T}}_{H},h_{2}({𝐗}_{t})=\gamma_{1}I_{t},h_{3}({𝐗}_{t})=\delta_{1}I_{t},h_{4}({𝐗}_{t})=\gamma_{2}A_{t},$ and $h_{5}({𝐗}_{t})=\delta_{2}A_{t}$.

Similarly as for the SIR process, we define the average realization for the SITAD process based the transmission matrix 𝐓 at time k as $E(I_{𝐓,k}^{+})=E(I_{𝐓,k}|I_{𝐓,k-1}=E(I_{𝐓,k-1}^{+}),T_{𝐓,k-1}=E(T_{𝐓,k-1}^{+}),A_{𝐓,k-1}=E(A_{𝐓,k-1}^{+}),D_{𝐓,k-1}=E(D_{𝐓,k-1}^{+}))$, $E(T_{𝐓,k}^{+})=E(T_{𝐓,k}|I_{𝐓,k-1}=E(I_{𝐓,k-1}^{+}),T_{𝐓,k-1}=E(T_{𝐓,k-1}^{+}),A_{𝐓,k-1}=E(A_{𝐓,k-1}^{+}),D_{𝐓,k-1}=E(D_{𝐓,k-1}^{+}))$, $E(A_{𝐓,k}^{+})=E(A_{𝐓,k}|I_{𝐓,k-1}=E(I_{𝐓,k-1}^{+}),T_{𝐓,k-1}=E(T_{𝐓,k-1}^{+}),A_{𝐓,k-1}=E(A_{𝐓,k-1}^{+}),D_{𝐓,k-1}=E(D_{𝐓,k-1}^{+}))$, $E(D_{𝐓,k}^{+})=E(D_{𝐓,k}|I_{𝐓,k-1}=E(I_{𝐓,k-1}^{+}),T_{𝐓,k-1}=E(T_{𝐓,k-1}^{+}),A_{𝐓,k-1}=E(A_{𝐓,k-1}^{+}),D_{𝐓,k-1}=E(D_{𝐓,k-1}^{+}))$. The average realization of the number of nodes in each state from time 1 to K in the network are $E(Y_{1}^{+}),E(Y_{2}^{+}),\cdots ,E(Y_{K}^{+}),$ where Y is one of the states S,I,T,A,D.

The following lemma tells us that the average realizations based on the two approaches are the same.

**Lemma 2.3** If the order of infections is known, the spreading process based on equation (6) has the same average realization as the SITAD process on the network.

### 2.3 Approximations of the spreading processes using the average transmission matrix

As shown in the preceding sections, when the infection sequence is known, the modified SI, SIR, and SITAD processes generate the same average number of infections on networks. Since the order of infections is often unknown, we can determine the order of infections using random sampling. We proved that when the infection order is known, the average realization of the number of infections based on the corresponding transmission matrix equals the average realization based on the network. As a result, the average number of infections based on transmission matrices ${𝐓}_{i},$ for i=1,⋯,m will converge to the average realization of the network spreading process as the sample size m grows.

For a given infection sequence with the transmission matrix ${𝐓}_{i}$, if the transmission rate is β, $\beta {𝐓}_{i}$ represents the matrix of average spreading rates corresponding to the infection sequence. Therefore, the average spreading rate corresponding to m different realizations ${𝐓}_{i},$ for i=1,⋯,m can be approximated by $\beta {𝐓}_{\text{avg}}$, where ${𝐓}_{\text{avg}}=1/m\sum_{i=1}^{m}{𝐓}_{i}$. In other words, the average number of infections based on the modified process utilizing the average transmission matrix ${𝐓}_{\text{avg}}$ can be used to approximate the average number of infections generated by the network spreading process. We refer to this approximation approach as the average transmission matrix model, or ATMM.

## 3. Results

In this Section, we provide results related to theoretical, simulation, and a quantitative answer for the usefulness of network information to study epidemics if such information is accessible. Section 3.1 provides some theoretical results of using the proposed method to study the reproduction number of the spreading process on networks. Section 3.2.1 shows that the modified spreading process agrees with the proposed network spreading process in terms of the average number of infections over time. In Section 3.2.2, we use synthetic network data to show how the modified spreading process outperforms the proposed spreading process in terms of computation. Finally, Section 3.3 demonstrates that using the network model to analyze network epidemic data, once network information is accessible, surpasses the mass-action model both in terms of computational efficiency and goodness of fit.

The synthetic data used in Sections 3.2.2 and 3.3 below are generated using the proposed discrete-time SIR process in Section 2.2.2 on an empirical network dataset. The empirical network data is an aggregate of network data obtained from the Copenhagen Network Study (CNS), which was made publicly accessible in 2019 [37]. The network data comprises the connectivity patterns of 706 students at the Technical University of Denmark during a 28-day period in February 2014. The connectivity patterns are identified through the use of Bluetooth as participants consented to use loaner phones provided by the study as their main phone throughout the study. The received signal strength indicator (RSSI), which can serve as an approximation of physical distance, was collected every five minutes. Following Hambridge et. al. [38], we assigned a connection between two persons if there was at least one RSSI signal large enough during the period, i.e., RSSI ≥−75dBm. For analysis purposes, we simply kept the largest component, which contained 673 nodes and 57,712 edges, as a fixed network. Based on the fixed empirical network, we synthesized epidemic data. For generating network epidemic data, we used the discrete time SIR proposed spreading process as described in Section 2.2.2. In particular, we first generated model parameters from prior distributions and then simulated network epidemic data using the generated parameters. If the synthesized data realization was good enough, meaning there was enough data to estimate model parameters, we kept it and retained the model parameters. Since there were only 673 nodes in the observed network, we specified that a good realization had to have cumulatively at least 50% of nodes infected and 10% of nodes recovered. Once these constraints were met, the synthesized data was treated as observed network epidemic data, with the corresponding parameters serving as the underlying truth to evaluate the accuracy of estimation.

### 3.1 Theoretical results on the early behavior of the proposed SIR spreading process on networks

In this section, we present an advantage of using the proposed method for studying epidemics on networks related to reproduction numbers. For simplicity, we consider the proposed SIR process on networks. Under this process, we will show that the proposed method has the merit of providing a straightforward derivation of the basic reproduction number $R_{0}$ and the reproduction number at the early stage $R_{t}$. Finally, we will provide a result on the epidemic behavior during the early stage for any network size.

From Lemma 2.2, we know that once the order of infections is known, the proposed SIR spreading process on a network has the same average realization as the modified spreading process based on the transmission matrix corresponding to the infection order as described in the system of equations (5). Therefore, we can study the basic reproduction number of the SIR process on a network by using the analog system of equations (5) in a similar manner as in the mass-action model.

We have $\frac{dI}{dt}=I\beta {\overset{―}{T}}_{H}-\gamma I=I(\beta {\overset{―}{T}}_{H}-\gamma )$. So the basic reproduction number $R_{0}$ of the spreading process on the network is $R_{0}=\frac{\beta }{\gamma }{\overset{―}{T}}_{1}$. Note that, when $R_{0}>1$, large outbreaks that are driven to extinction by depletion of susceptibles occur with positive probability. Let us denote the sequence of distinct node degrees of the given network as $\left\{k_{1},\cdots ,k_{s}\right\}$; the probability a given node has degree $k_{i}$ is $p_{i}$, where $\sum_{i=1}^{s}p_{i}=1$ and 1≤s≤N. If the initial infected node is node i, then the basic reproduction number $R_{0}$ is $R_{0}=\frac{\beta }{\gamma }{\overset{―}{T}}_{1}=\frac{\beta }{\gamma }\frac{k_{i}}{k_{i}+1}=\frac{\beta }{\gamma }(1-\frac{1}{k_{i}+1})$. If the initially infected node is unknown, the basic reproduction number now follows a distribution induced by the node degree distribution where $R_{0}=\frac{\beta }{\gamma }(1-\frac{1}{k_{i}+1})$ with probability $p_{i}$, where $p_{i}$ is again the probability a node has degree $k_{i}$. The average basic reproduction number is $\overset{―}{R_{0}}=\sum_{i=1}^{s}p_{i}\frac{\beta }{\gamma }(1-\frac{1}{k_{i}+1})=\frac{\beta }{\gamma }(1-\sum_{i=1}^{s}p_{i}\frac{1}{k_{i}+1})$.

Denote $k^{*}=\text{min}\{k_{1},\cdots ,k_{s}\}$. We have the following bounds: $\frac{\beta }{\gamma }(1-\frac{1}{k^{*}+1})<\frac{\beta }{\gamma }(1-\frac{1}{k_{i}+1})<\frac{\beta }{\gamma }(1-\frac{1}{N})$ for all i∈1,⋯,k. Since the network structure underlying the mass action model is a fully connected network, its basic reproduction number is $R_{0}^{c}=\frac{\beta }{\gamma }(\frac{N-1}{N})=\frac{\beta }{\gamma }(1-\frac{1}{N})$. We see that the quantity $R_{0}^{c}$ is an upper bound on the basic reproduction number of a spreading process on a network. This tells us that if the process starts with one initially infected node, the spreading process on a network will less likely lead to an epidemic compared to the mass-action model. The lower bound of the above inequality tells us that the epidemic will least likely occur if the initially infected node has the smallest number of neighbors (smallest degree).

Next, we consider the early stage behavior of the effective reproduction number $R_{t}$, for t small. For simplicity, suppose that at time t, the network has h infected nodes and no recovered nodes. Let I denote the set of h infected nodes at time t, we have $R_{t}=\frac{\beta }{\gamma }{\overset{―}{T}}_{h}=\frac{\beta }{\gamma }\frac{1}{h}\sum_{i\in \text{I}}\frac{S_{i}}{k_{i}+1}$. First, we look at the upper bound and lower bound of the quantity $\frac{S_{i}}{k_{i}+1}$ at each infected node i. Since in a network with h infected nodes, node i is one of them, and there are h−1 other infected nodes. The number of susceptible neighbors of node i, $S_{i}$, depends on the positions of the other h−1 infected nodes. In one extreme case, if only one of node i’s neighbors is infected and the remaining h−2 infected nodes are not neighbors of node i, then $S_{i}$ reaches its maximum value, which is $k_{i}-1$. On the other hand, if all h−1 infected nodes are neighbors of node i, then the number of susceptible neighbors of node i is reduced to $k_{i}-(h-1)$. Therefore, we can establish the following inequality: $k_{i}-(h-1)\leq S_{i}\leq k_{i}-1.$ Dividing through by $k_{i}+1$, we obtain $1-\frac{h}{k_{i}+1}\leq \frac{S_{i}}{k_{i}+1}\leq 1-\frac{2}{k_{i}+1}.$ Therefore, the lower bound $R_{t}^{L}$ and upper bound $R_{t}^{U}$ of $R_{t}$ are given by

$R_{t}^{L}=\frac{\beta }{\gamma }(1-\sum_{i\in \text{I}}\frac{1}{k_{i}+1})\leq R_{t}\leq R_{t}^{U}=\frac{\beta }{\gamma }(1-\frac{2}{h}\sum_{i\in \text{I}}\frac{1}{k_{i}+1})$. We observe that the effective reproduction number of the spreading process on networks attains its upper bound if the spreading path of h infected nodes form a line, and it attains its lower bound if the spreading path of h infected nodes forms a complete graph of h nodes.

Finally, we consider the important question of whether there are any scenarios where the spreading process on a network is more aggressive than the mass-action model (where its corresponding network structure is fully connected). The following Proposition gives us the answer to this important question.

**Proposition 3.1** Consider the SIR proposed spreading process on networks at the early stage with h infected nodes.

a. For large networks as size N→∞, the asymptotic behavior of the effective reproduction number $R_{t}$ at the early stage of the epidemic on networks is always asymptotically bounded above by the effective reproduction number of the mass-action model.
b. For finite-size networks, the effective reproduction number $R_{t}$ at the early stage of the epidemic on networks is greater for the mass-action model if all h infected nodes form a chain graph and each infected node has more than 2N/h−1 susceptible neighbors.

Proposition 3.1 shows that for a large network, its effective reproduction number $R_{t}$ at the early stage (t small) is always asymptotically bounded from above by its counterpart mass-action model. However, given a finite-size network, the network spreading process can be more aggressive than the mass-action model depending on the spreading pattern and network topology. This highlights the importance of network topology in understanding disease dynamics.

**Fig 4:**
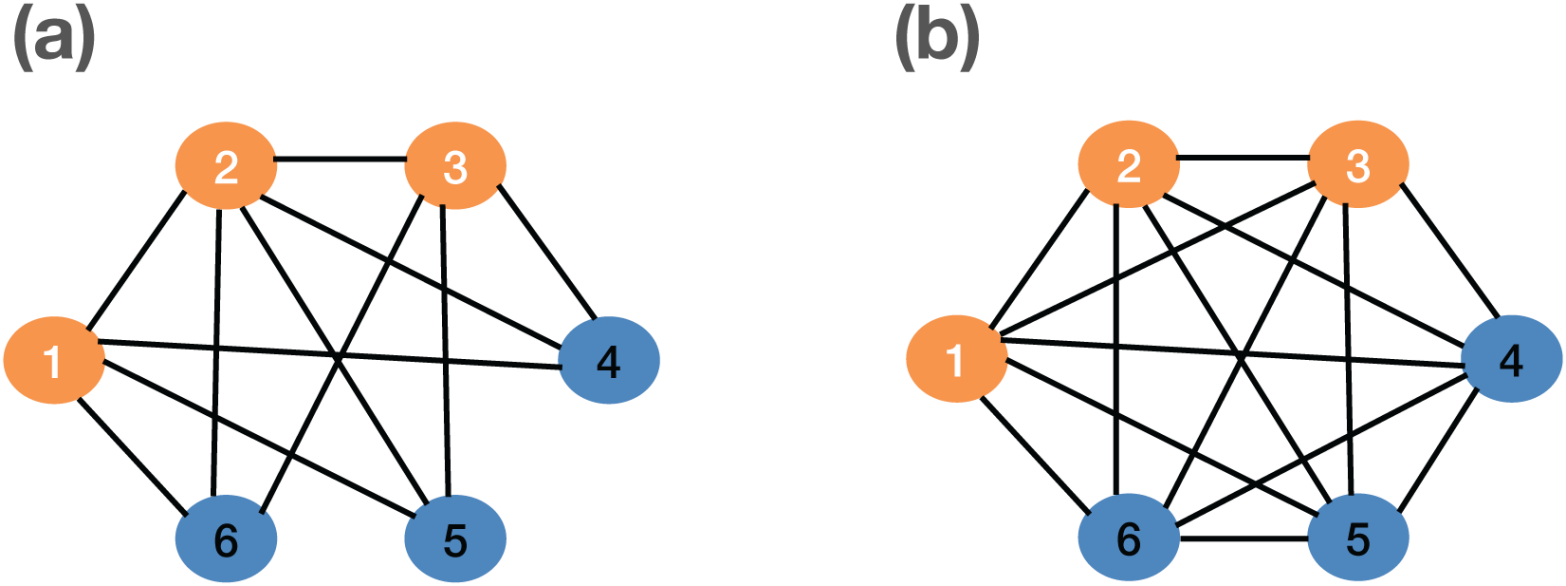
Comparing transmission rates of two networks with three infected nodes (1,2,3): (a) partially connected network with the total transmission rate of 2.1, and (b) fully connected network with the total transmission rate of 1.5.

Fig 4 demonstrates the case where the spreading process on a partially connected network is more aggressive than the spreading process on a fully connected network. In this case, the infected nodes on the partially connected network as in Fig 4a form a chain graph. The total transmission rate on the partially connected network is 2.1, while the total transmission rate on the fully connected network is 1.5.

### 3.2 Simulation results on the proposed spreading process and its modifications

#### 3.2.1 Modified spreading processes.

**Fig 5:**
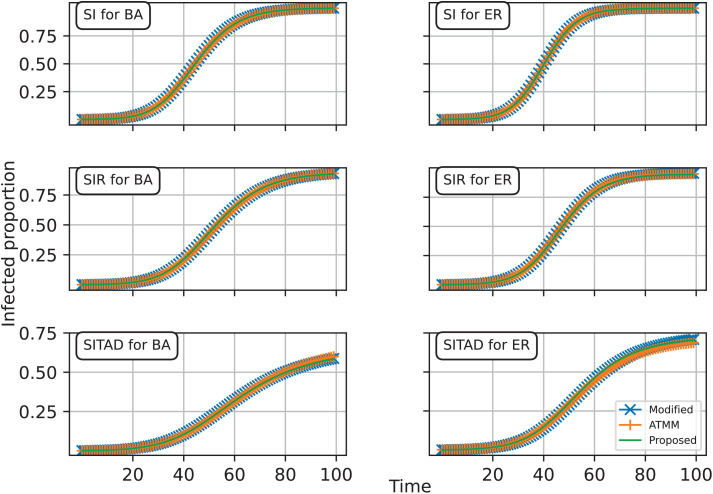
Approximation of different approaches for the SI, SIR, and SITAD spreading processes on the BA network (left) and modified ER network with a single connected component (right). The first row corresponds to the SI process with the model parameter θ=0.2, the second row to the SIR process with θ=(0.18,0.01), and the last row to the SITAD process with θ=(0.2,0.1,0.05,0.03,0.02,0.01).

We conducted simulation studies for the three processes discussed in the paper: SI, SIR, and SITAD. For each process, the fixed network structure was generated from a network model. We considered networks of N=1000 nodes generated by the Erdős–Rényi (ER) model with the probability parameter p=0.1, and the Barabási–Albert (BA) model with the parameter m=15. Note that the parameters p and m in the ER and BA models influence the network’s density. The larger of these values, the network is denser. While p is the probability that two random nodes among N nodes are connected, m refers to the number of edges a new coming node will create to connect with existing nodes in the network. Since the ER network can have multiple components, we forced it to one component by adding the set of edges {(1,2),(2,3),⋯,(N−1,N}. Without loss of generality, we assumed that node 1 is the initial infected node. Based on the given network, the initially infected node, and the model parameter θ, the average number of infections is determined by averaging the number of infections resulting from 1000 iterations of disease transmission using the proposed spreading rule on the network. On the other hand, using the sampling procedure, we generated 30 infection order sequences and their corresponding transmission matrices ${𝐓}_{i}$, for i=1,⋯,30. Then, we used the modified process that utilized random transmission matrices $\{{𝐓}_{i}{\}}_{i=1,\cdots ,30}$ to produce 1000 realizations of the number of infections. We obtained the average realization of the number of infections of the modified process by taking the average of these infection sequences. We also considered the ATMM by applying the modified process to the average transmission matrix ${𝐓}_{\text{avg}}=\frac{1}{30}\sum_{i=1}^{30}{𝐓}_{i}$. In particular, we simulated the modified process utilizing the average transmission matrix 1000 times. We then calculated the average number of infections by averaging those 1000 simulated realizations. Fig 5 shows a good agreement across the different approaches.

#### 3.2.2 Proposed SIR model and the SIR ATMM.

In this Section, we use synthetic network data to illustrate the benefits of the modified spreading process in estimating model parameters. In particular, we present a comparison of the performance of the proposed SIR process and the SIR ATMM in estimating model parameters based on observed network data. To estimate the model parameters, we employ approximate Bayesian computation (ABC), a method that bypasses the need for a direct likelihood calculation. There are many variants of ABC, but they are all based on a comparison of observed and simulated data. The key idea of ABC is to start by sampling parameter values from prior distributions, then use the model and these parameter values to generate a data realization. The sampled parameter value is retained if its distance to the observed data is close enough. The collection of accepted parameter values constitutes a sample from an approximation of the posterior distribution and is used to estimate the model parameters. The variant ABC method used in this paper is replenishment ABC (RABC) [39]. Criteria for comparison include computational time, confidence interval coverage probability from posteriors, and interquartile range. To obtain these metrics, we setup the code as follows.

*Step 1. Generating data and parameters*: For i∈{1,⋯,100}, we generate the parameter $\theta^{\left(i\right)}=(\beta^{\left(i\right)},\gamma^{\left(i\right)})$ from uniform priors $\beta^{\left(i\right)}\sim U(0,\mathrm{.3})$ and $\gamma^{\left(i\right)}\sim U(0,\mathrm{.2})$. Based on the parameters, empirical network, and the proposed SIR model, we generate a data set ${\text{Data}}^{\left(i\right)}$ corresponding to $\theta^{\left(i\right)}$. If the generated data set ${\text{Data}}^{\left(i\right)}$ constitutes a good realization as defined above, we keep $\theta^{\left(i\right)}$ as a true parameter value to be estimated and treat the generated data $\left\{S_{t}^{\left(i\right)},I_{t}^{\left(i\right)},R_{t}^{\left(i\right)}\right\}$ as observed data. We repeat the process until we obtain 100 underlying true parameter values $\theta^{\left(i\right)}$ and the corresponding 100 datasets $\left\{S_{t}^{\left(i\right)},I_{t}^{\left(i\right)},R_{t}^{\left(i\right)}\right\}$. For simplicity, we fix the initial infected node at node 1 and set the simulation time period T=100 for all i.

*Step 2. Estimating parameters*: For each iteration i, i∈{1,⋯,100}, based on the sequence of $\left\{S_{t}^{\left(i\right)},I_{t}^{\left(i\right)},R_{t}^{\left(i\right)}\right\}$, we use RABC to estimate the underlying true parameter value $\theta^{\left(i\right)}$. In this estimation step, we chose priors for β as U(0,1), γ as U(0,.5), the final threshold as 40, and sampled 100 particles to form the posterior. We also used the simple Euclidean distance, $\text{D}=\sqrt{\sum_{t=1}^{99}(I(t)-I^{\left(s\right)}(t){)}^{2}}+$ $\sqrt{\sum_{t=1}^{99}((R(t)-R^{\left(s\right)}(t){)}^{2}}$, where t=1,…,99 are the days during the study period, I(t) is the number of infected nodes and R(t) is the number of recovered nodes at time t; $I^{\left(s\right)}(t)$ and $R^{\left(s\right)}(t)$ are the corresponding numbers from simulated data.

*Step 3. Evaluating parameter estimates*: For each synthesized data set i, i∈{1,⋯,100}, we evaluated the accuracy of our parameter estimates for each method based on coverage probability of the interquartile (IQ Cover), coverage probability of the 95 percentile interval (95% Cover), and the average interquartile range (IQR = $Q_{3}-Q_{1}$), for each parameter $\beta^{\left(i\right)},\gamma^{\left(i\right)}$. We then calculate the average of these 100 IQ Cover, 95% Cover, and IQR. We also compared the average time requirements to obtain the estimators corresponding to each realization using each method. The computation time is based on the results after submitting the parallel Python code to the University of Tennessee of Chattanooga Cluster with 3GB of memory and one CPU per task.

Table 1 presents the averages of running time, IQ Cover, 95% Cover, and IQR when using ABC to estimate model parameters with the Proposed SIR and SIR ATMM. The reported credible intervals show slight overcoverage, which is a known and generally acceptable feature of ABC methods, especially under low tolerance thresholds, and reflects the method’s conservative approach to uncertainty [40]. Beyond this, the table shows that the average transmission matrix model estimated parameters with comparable accuracy to the proposed model, while significantly reducing computation time (from 13.7 hours to 0.4 hours on average).

**Table 1:** Comparison of parameter estimation between the proposed SIR model and the SIR ATMM using RABC.

Method	Average time (h)	Parameter	IQ Cover	95% Cover	IQR
Proposed SIR	13.7	*β*	0.474	0.982	0.043
		*γ*	0.684	1.000	0.028
SIR ATMM	0.4	*β*	0.456	0.965	0.042
		*γ*	0.702	1.000	0.027

This improvement was to be expected as the average transmission matrix model requires network information in order to obtain the transmission matrix; once the transmission matrix is available, the spreading process can proceed at the same rate as the mass-action model. This important aspect addresses a significant challenge associated with the utilization of ABC in network infectious disease epidemiology research: the lengthy computational time required to directly simulate epidemic data on network for calibration purposes. The average transmission matrix approach is thus an excellent candidate for implementing ABC in network epidemiology.

### 3.3 Real data analysis on the necessity of network information in studying epidemics

Here, we address a fundamental question: If network information were readily accessible, how useful would it be compared with merely using the mass-action model? We provide a quantitative answer to this question by comparing the fit of the average transmission matrix model and the mass-action model to observed data, as well as the computation time required for each method. Specifically, we initially utilized ABC to estimate the model parameters for each model based on the observed data. We then used the ABC posteriors of model parameters to find the average realization and the 95% confidence band for the number of current and cumulative infected cases (infected and recovered) for every approach.

We calculated the 95% confidence interval for each approach as follows. We simulated three distinct SIR data sets using each model parameter sample of the ABC posteriors, and we retained only the best 30 simulated data that were closest to the observed data. The point of simulating three data sets for each model parameter is to avoid losing the particle (posterior sample) by chance, as the spreading process on a network might cause the realization to stop abruptly if the recovered nodes are in bottleneck positions at the early stages of the spreading process. From the best 30 realizations, we constructed a 95% confidence interval for each method. In addition, for each of the 30 realizations, we calculated the Euclidean distance as defined in Section 3.2.2. Based on these distances, we calculated the mean distance and its standard deviation.

**Fig 6:**
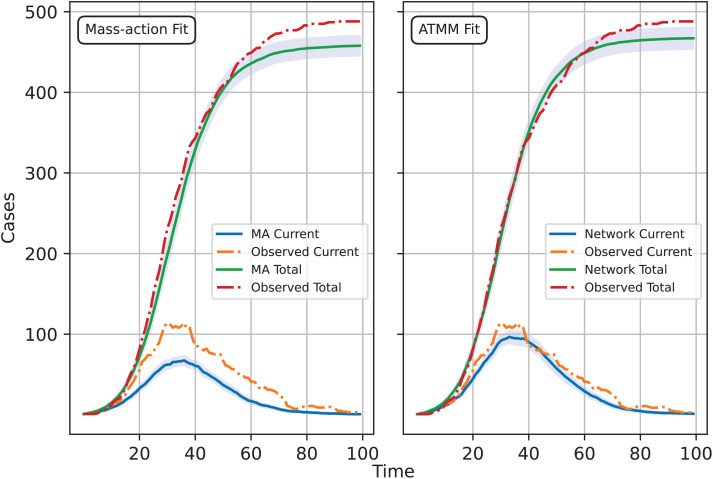
Comparison of the 95% confidence band for the mass-action model and the ATMM.

Fig 6 demonstrates that network information provides a far better fit to the observed data. The 95% confidence band derived from the average transmission matrix method effectively captures the observed data. However, when naively applying the mass-action model to fit the spreading process on the network, the results deviate significantly from the observed data. Table 2 provides more information on the distance and time for each model. Here, we take the final threshold as 40 for the ABC procedure. The table shows that adopting the mass-action model to match the epidemic data naively not only results in a worse fit than the average transmission matrix model but also requires a threefold increase in processing time. This interesting phenomenon arises because the computer is having difficulty finding a suitable fit between data generated by the mass-action model and the observed network data. If the ABC acceptance threshold is lower than 40, the mass-action model will eventually fail to converge because we are using a wrong model to fit the network epidemic. Therefore, network knowledge is extremely valuable and can provide insights into the nature of epidemics.

**Table 2:** Comparison of the mass-action and the naive method.

Method	Time (h)	Mean Distance	Std Distance
Mass-action	2.1	46.593	2.770
ATMM	0.7	44.566	1.783

## 4. Discussion

In this study, we examined the connection between network models and mass-action models. We proposed a spreading rule on networks that allows for an exact match between the epidemic spread on the network and the classic mass-action models when the graph is fully connected. We then developed modified spreading processes on networks that are similar to the classic mass-action models. We also proved that the modified processes and the proposed spreading rule on networks have the same average number of infections. Our results reveal some of the differences between the two models as well as how the network model differs from the traditional mass-action model. More specifically, we noted that the variety of spreading rules in the network-based model cause it to differ from its mass-action counterpart. The proposed spreading rule allows us to bridge the two models as it shares a similar underlying spreading mechanism with the mass-action model. When the network is fully connected, the proposed rule and the mass-action model align regardless of population size. However, when the network is partially connected, for a given infection order, the spreading process on network and mass-action models diverges due to the different transmission matrices driving each model.

Besides considering the popular SI and SIR spreading processes on networks, we extended the SITAD model to networks. We also analyzed and compared outbreaks during the early stage of the SIR spreading process for network and mass-action models. By utilizing synthesized data from an empirical network, we highlighted the benefits of network information in studying epidemics and the advantages of the proposed method. In particular, we demonstrate that the network structure is crucial for improving the fit. Additionally, we show that the modified version of our approach, ATMM, is computationally efficient with little loss of accuracy.

Furthermore, our approach allows us to transform epidemic spread on arbitrary networks into equivalent mass-action models. When employing simulation-based inference, which typically requires extensive calibration datasets, this transformation enables us to estimate model parameters for network epidemics more efficiently (especially for large networks): instead of repeatedly simulating the spreading process on the network, we can generate calibration data faster from the corresponding mass-action model.

The main limitation of the modified process is that the network is fixed. In practice, the network may evolve over time. There are also many extensions that can be conducted using the proposed spreading process, such as investigating different epidemiological quantities such as the basic reproduction number $R_{0}$, the exact/approximate solution of the spreading process, and prevention strategies on the network.

Finally, over the past couple of decades, research in network science has provided valuable insights into epidemics. Centrality measures and clustering have proven highly effective in identifying high-risk groups and optimizing intervention strategies [9]. As global connectivity and emerging diseases continue to challenge public health systems, the ability to model and analyze disease transmission through network-based approaches will be increasingly crucial in addressing evolving infectious disease threats.

## Supporting information

S1 File Proofs for Lemmas 2.1, 2.2, and 2.3; Proposition 3.1; Pseudocode for commonly used network spreading algorithms: SI Gillespie, SI unit infectivity, SI degree infectivity, the proposed SI, SIR, SITAD spreading rules, and the sampling process for determining infection order sequence. **Algorithm 1**: The Gillespie SI spreading rule. **Algorithm 2**: The unit infectivity SI spreading rule. **Algorithm 3**: The degree infectivity SI spreading rule. **Algorithm 4**: The proposed SI spreading rule on networks. **Algorithm 5**: The proposed SIR spreading rule on networks. **Algorithm 6**: The proposed SITAD spreading rule on networks. **Algorithm 7**: The sampling mechanism to obtain the infection order sequence.
(PDF)
