# Supplementary material for: Connecting mass-action models and network models for infectious diseases
Source: PLoS Comput Biol. 2025 Aug 18;21(8):e1013373. doi: 10.1371/journal.pcbi.1013373 (PMC12416853; doi:10.1371/journal.pcbi.1013373)
Supplement: S1 File — Algorithm 1: The Gillespie SI spreading rule. Algorithm 2: The unit infectivity SI spreading rule. Algorithm 3: The degree infectivity SI spreading rule. Algorithm 4: The proposed SI spreading rule on networks. Algorithm 5: The proposed SIR spreading rule on networks. Algorithm 6: The proposed SITAD spreading rule on networks. Algorithm 7: The sampling mechanism to obtain the infection order sequence. (PDF) [file pcbi.1013373.s001.pdf]

Supplementary Information for: Connecting mass-action models and network models for infectious diseases

Thien-Minh Le[1] and Jukka-Pekka Onnela[2,*]

1 Department of Mathematics, The University of Tennessee at Chattanooga, Chattanooga, Tennessee, U.S.A.
2 Department of Biostatistics, Harvard T.H. Chan School of Public Health, Boston, Massachusetts, U.S.A.

*Corresponding author: onnela@hsph.harvard.edu

The Supplementary Information includes the following:

1. Proofs of Lemmas 2.1, 2.2., 2.3., Proposition 3.1. in the main text.
2. Pseudo-code for some commonly used network spreading algorithms: SI Gillespie, SI unit infectivity, SI degree infectivity.
3. Pseudo-code for the proposed SI, SIR, SITAD spreading rules, and the sampling process for determining infection order sequence.

Proof of Lemma 2.1.: Lemma 2.1. is a direct result from Lemma 2.2. Notice that if we replace the Poisson distribution with the Binomial distribution, the number of infections of the two approaches give rise to the same distribution. Q.E.D.
Proof of Lemma 2.2:

We need to prove that

$$E\big(I^+_{\mathbf{T},k}\big) = E\big(I^+_k\big), E\big(R^+_{\mathbf{T},k}\big) = E\big(R^+_k\big) \tag{1}$$

for all $k = 1, 2, \cdots$. We use induction to prove (1). For $k = 1$, on a given transmission matrix $\mathbf{T}$, we have
$E\big(I^+_{\mathbf{T},1}\big) = E\big(I_{\mathbf{T},1}|I_{\mathbf{T},0} = 1, R_{\mathbf{T},0} = 0\big) = E\big(I_{\mathbf{T}}(0)\big)+E\big(I_{\mathbf{T}}(0)\big)\beta\bar{T}_1-\gamma I_{\mathbf{T}}(0) = 1+\beta\bar{T}_1-\gamma$, where $\bar{T}_1 = \sum_{i=1}^N \mathbf{T}[1, i]/1 = \sum_{i=1}^N \mathbf{T}[1, i]$. On the other hand, the transmission on the network gives us:
$E\big(I^+(1)\big) = E\big(I(1)|I(0) = 1, R(0) = 0\big) = 1 + \sum_{j\in I(0)} S_j/(N_j + 1) - \gamma$. Since the infection order is known, $\sum_{j\in I(0)} S_j/(N_j + 1) = \sum_{i=1}^N \mathbf{T}[1, i] = \bar{T}_1$. Therefore,
$E\big(I^+(1)\big) = E\big(I^+_{\mathbf{T}}(1)\big)$. Suppose that (1) holds up to k, i.e.,
$E\big(I^+(k)\big) = E\big(I^+_{\mathbf{T}}(k)\big), E\big(R^+(k)\big) = E\big(R^+_{\mathbf{T}}(k)\big)$, we need to prove that (1) also hold for $(k + 1)$, i.e., we need to prove that
$E\big(I^+(k + 1)\big) = E\big(I^+_{\mathbf{T}}(k + 1)\big), E\big(R^+(k + 1)\big) = E\big(R^+_{\mathbf{T}}(k + 1)\big)$. We have
$E\big(R^+_{\mathbf{T}}(k + 1)\big) = E\big(R^+_{\mathbf{T}}(k)\big) + \gamma E\big(I^+_{\mathbf{T}}(k)\big)$, $E\big(R^+(k + 1)\big) = E\big(R^+(k)\big) + \gamma E\big(I^+_{\mathbf{T}}(k)\big)$.
From the induction hypothesis, we have $E\big(R^+_{\mathbf{T}}(k)\big) = E\big(R^+(k)\big)$ and
$E\big(I^+_{\mathbf{T}}(k)\big) = E\big(I^+_{\mathbf{T}}(k)\big)$, therefore $E\big(R^+_{\mathbf{T}}(k + 1)\big) = E\big(R^+(k + 1)\big)$. Finally, we need to prove that $E\big(I^+_{\mathbf{T}}(k + 1)\big) = E\big(I^+(k + 1)\big)$. Since $E\big(I^+_{\mathbf{T}}(k + 1)\big) = E\big(I_{\mathbf{T}}(k + 1)|I_{\mathbf{T}}(k) = E\big(I^+_{\mathbf{T}}(k)\big), R_{\mathbf{T}}(k) = E\big(R^+_{\mathbf{T}}(k)\big)\big) = E\big(I^+_{\mathbf{T}}(k)\big) + E\big(I^+_{\mathbf{T}}(k)\big)\beta\bar{T}_{H_{\mathbf{T}}(k)} - \gamma E\big(I^+_{\mathbf{T}}(k)\big)$, where $H_{\mathbf{T}}(k) = E\big(I^+_{\mathbf{T}}(k) + R^+_{\mathbf{T}}(k)\big)$. On the other hand, we have

$E\big(I^+(k+1)\big) = E\big(I(k+1)|I(k) = E\big(I^+(k)\big), R(k) = E\big(R^+(k)\big)\big) =$
$E\big(I^+(k)\big) + E\big(I^+(k)\big)\beta\big(\sum_{j\in\mathbf{H}(k)} S_j/(N_j+1)\big)/H(k) - \gamma E\big(I^+(k)\big)$, where $\mathbf{H}(k)$ is the set of all currently infected nodes and $H(k) = E\big(I^+(k) + R^+(k)\big)$. By the induction hypothesis, we have $E\big(I^+(k)\big) = E\big(I^+_{\mathbf{T}}(k)\big), E\big(R^+(k)\big) = E\big(R^+_{\mathbf{T}}(k)\big)$, therefore $H(k) = H_{\mathbf{T}}(k)$. Since the infection order is known, $\sum_{j\in\mathbf{H}(k)} S_j/(N_j+1) = H(k)\bar{T}_{H(k)} = H(k)\bar{T}_{H_{\mathbf{T}}(k)}$. So,
$E\big(I^+(k+1)\big) = E\big(I^+(k)\big) + E\big(I^+(k)\big)\beta\bar{T}_{H_{\mathbf{T}}(k)} - \gamma E\big(I^+(k)\big) =$
$E\big(I^+_{\mathbf{T}}(k)\big) + E\big(I^+_{\mathbf{T}}(k)\big)\beta\bar{T}_{H_{\mathbf{T}}(k)} - \gamma E\big(I^+_{\mathbf{T}}(k)\big) = E\big(I^+_{\mathbf{T}}(k+1)\big)$. The lemma is proved.
Proof of Lemma 2.3.: Repeat the argument as in Lemma 2.2.
Proof of Proposition 3.1.:

a. The effective reproductive number for the mass-action model when it has h infected nodes is $R^C_t = \frac{\beta}{\gamma}(1 - \frac{h}{N}) \xrightarrow{N\to\infty} \frac{\beta}{\gamma}$. We also have $R^U_t = \frac{\beta}{\gamma}(1 - \frac{2}{h}\sum_{i\in\mathcal{I}} \frac{1}{k_i+1}) =\leq \frac{\beta}{\gamma}(1 - \frac{2}{N}) \xrightarrow{N\to\infty} \frac{\beta}{\gamma}$. Therefore, for large network with $N \to \infty$, at the early stage, the effective reproductive number R_t on the network is always asymptotically bounded by the effective reproductive number of the mass action model.

b. Let us denote $k^*_{\mathcal{I}} = \min\{k_i\}_{i\in\mathcal{I}}$. The upper bound and lower bound of R^U_t are given by $\frac{\beta}{\gamma}(1 - \frac{2}{k^*_{\mathcal{I}}+1}) \leq R^U_t \leq \frac{\beta}{\gamma}(1 - \frac{2}{N})$. We also observe that that $R^C_t = \frac{\beta}{\gamma}(1 - \frac{h}{N}) \leq \frac{\beta}{\gamma}(1 - \frac{2}{k^*_{\mathcal{I}}+1})$ iff $k^*_{\mathcal{I}} \geq \frac{2N}{h} - 1$. So, for finite networks of size N with h infected nodes, if the spreading process of h nodes occurred in a line graph and each infected node has more than $\frac{2N}{h} - 1$ neighbors, the epidemic on the network at that time is more aggressive than the epidemic on the mass-action model.

Algorithms 1-7 below provide the pseudo-codes for different spreading rules and procedures mentioned in the main text. More specifically, Algorithms 1-3 are the pseudo-code for the commonly spreading rules used in practice, including the Gillespie, the unit infectivity, and the degree infectivity; Algorithms 4-6 are the pseudo-codes for the proposed spreading rules for the SI, SIR, and SITAD processes; and Algorithm 7 is the pseudo-code for the sampling procedure to obtain the infection order sequence.

Algorithm 1 The Gillespie SI spreading rule

```
Input: G, per-edge transmission rate τ, initial_I, max time T.
Output: List times, # of S, I at each time

function GillespieSI_network_spread(G, τ, T, initial_I)
  time, S, I ← [0], [|G| - len(initial_I)], [len(initial_I)]
  I_nodes, S_nodes ← initial_I, G \ initial_I
  at_risk_nodes ← S neighbors of I
  for each node u in at_risk_nodes do
      infection_rate[u] = τ× # of I neighbors
  total_infection_rate = Σ_{u∈at_risk_nodes} infection_rate[u]
  time ← Exp_variate(total_infection_rate)
  while time < T do
      p_u = infection_rate[u]/total_infection_rate
      choose u from at_risk_nodes with probability p_u
      remove u from at_risk_nodes
      add u to I_nodes
      for S neighbors v of u do
        if v not in at_risk_nodes then
          add v in at_risk_nodes
        update infection_rate[v]
      update times, S_nodes, I_nodes, S, I
      time ← time + Exp_variate(total_infection_rate)
    return times, S, I
```

Algorithm 2 The unit infectivity SI spreading rule

```
Input: G, per-edge transmission rate τ, initial_I, max time T.
Output: # of S, I at each time

function UnitInfectivitySI_network_spread(G, τ, T, initial_I)
  time, S, I ← 0, [|G| - len(initial_I)], [len(initial_I)]
  I_nodes, S_nodes ← initial_I, G \ initial_I
  while time < T do
    for each node u in I_nodes do
      randomly choose a neighbor v of u
      if v is S then
          add v to I_nodes with probability τ
    update S_nodes, I_nodes, S, I
    time ← time+ 1
  return S, I
```

Algorithm 3 The degree infectivity SI spreading rule

Input: G, per-edge transmission rate τ, initial_I, max time T.
Output: # of S, I at each time

```
function DegreeInfectivitySI_network_spread(G, τ, T, initial_I)
    time, S, I ← 0, [|G| - len(initial_I)], [len(initial_I)]
    I_nodes, S_nodes ← initial_I, G \ initial_I
    while time < T do
        for each node u in I_nodes do
            for each neighbor v of u do
                if v is S then
                        add v to I_nodes with probability τ
        update S_nodes, I_nodes, S, I
        time ← time + 1
    return S, I
```

Algorithm 4 The proposed SI spreading rule on networks

Input: G, transmission rate β, initial_I, max time T.
Output: # of S, I at each time

```
function ProposedSI_network_spread(G, β, T, initial_I)
    time, S, I ← 0, [|G| - len(initial_I)], [len(initial_I)]
    I_nodes, S_nodes ← initial_I, G \ initial_I
    at_risk_nodes ← S neighbors of I
    while time < T do
        for each node u in at_risk_nodes do
                update W[u] = # I neighbors of u
        for each node v in I_nodes do
            I_rate[v] = S neighbors of v/(neighbors of v + 1)
            at_risk_nodes ← at_risk_nodes + S neighbors of v
        transmission_rate = β× sum(I_rate)
        new_I ← Binom(at_risk_nodes, transmission_rate/ at_risk_nodes)
        if new_I > 0 do
            sample new_I nodes from at_risk_nodes with weight W
        update S_nodes, I_nodes, at_risk_nodes, S, I
        time ← time + 1
    return S, I
```

Algorithm 5 The proposed SIR spreading rule on networks

```
Input: G, θ = (β, γ), initial_I, max time T.
Output: # of S, I, R at each time

  function ProposedSIR_network_spread(G, θ, T, initial_I)
    time, S, I, R ← 0, [|G| - len(initial_I)], [len(initial_I)], [0]
    I_nodes, S_nodes ← initial_I, G \ initial_I
    at_risk_nodes ← S neighbors of I
    R nodes ← [ ]
    while time < T do
      for each node u in at_risk_nodes do
          update W[u] = # I neighbors of u
      for each node v in I_nodes do
        I_rate[v] = S neighbors of v/(neighbors of v + 1)
        at_risk_nodes ← at_risk_nodes + S neighbors of v
      transmission_rate = β× sum(I_rate)
      new_I ← Binom(at_risk_nodes, transmission_rate/ at_risk_nodes)
      new_R ← Binom(# of I_nodes, γ)
      if new_I > 0 do
        sample new_I nodes from at_risk_nodes with weight W
      randomly choose new_R nodes from I_nodes
      update S_nodes, I_nodes, R nodes, at_risk_nodes, S, I, R
      time ← time + 1
    return S, I, R
```

Algorithm 6 The proposed SITAD spreading rule on networks

```
Input: G, θ = (β1, β2, γ1, δ1, γ2, δ2), initial_I, max time T.
Output: # of S, I, T, A, D at each time
  function ProposedSITAD_network_spread(G, θ, T, initial_I)
    time, S, I, T, A, D ← 0, [|G| - len(initial_I)], [len(initial_I)], [0], [0], [0]
    I_nodes, S_nodes ← initial_I, G \ initial_I
    T_nodes ← [ ]
    A_nodes ← [ ]
    D_nodes ← [ ]
    at_risk_nodes ← S neighbors of I_nodes and A_nodes
    while time < T do
      for each node u in at_risk_nodes do
          W[u] = β1× # I neighbors of u + β2× # A neighbors of u
      for each node v1 in I_nodes do
        I_rate[v1] = S neighbors of v1/(neighbors of v1 + 1)
        at_risk_nodes ← at_risk_nodes + S neighbors of v1
      for each node v2 in A_nodes do
        A_rate[v2] = S neighbors of v2/(neighbors of v2 + 1)
        at_risk_nodes ← at_risk_nodes + S neighbors of v2
      transmission_rate = β1× sum(I_rate) + β2× sum(A_rate)
      new_I ← Binom(at_risk_nodes, transmission_rate/ at_risk_nodes)
      I_treated ← Binom(# of I_nodes, γ1)
      A_treated ← Binom(# of A_nodes, γ2)
      new_T← I_treated + A_treated
      if new_I > 0 do
        sample new_I nodes from at_risk_nodes with weight W
      randomly choose I_treated nodes from I_nodes
      randomly choose A_treated nodes from A_nodes
      new_A ← Binom(# of I_nodes, δ1)
      new_D ← Binom(# of A_nodes, δ2)
      randomly choose new_A_nodes from I_nodes
      randomly choose new_D_nodes from A_nodes
      update S_nodes, I_nodes, T_nodes, A_nodes, D_nodes, at_risk_nodes
      update S, I, T, A, D
      time ← time + 1
    return S, I, T, A, D
```

Algorithm 7 The sampling mechanism to obtain the infection order sequence

```
Input: G, initial_I
Output: The infection order sequence

  function Infection_order(G, initial_I)
    I_nodes, S_nodes ← initial_I, G \ initial_I
    at_risk_nodes ← S neighbors of I
    I_order_sequence ← initial_I
      for each node u in at_risk_nodes do
          update W[u] = # of I neighbors
        sample 1 new I node from at_risk_nodes with weight W
      update S_nodes, I_nodes, I_order_sequence
    return I_order_sequence
```
